# DeepVaR: a framework for portfolio risk assessment leveraging probabilistic deep neural networks

**DOI:** 10.1007/s42521-022-00050-0

**Published:** 2022-04-13

**Authors:** Georgios Fatouros, Georgios Makridis, Dimitrios Kotios, John Soldatos, Michael Filippakis, Dimosthenis Kyriazis

**Affiliations:** 1 Department of Digital Systems, University of Piraeus, Karaoli and Dimitriou 80, 18534 Piraeus, Greece; 2 Innov-Acts Ltd, Kolokotroni 6, 1101 Nicosia, Cyprus

**Keywords:** Probabilistic deep neural networks, Time-series, Forex, Finance, VaR, Risk assessment, VaR prediction, C22, C45, C53, C63

## Abstract

Determining and minimizing risk exposure pose one of the biggest challenges in the financial industry as an environment with multiple factors that affect (non-)identified risks and the corresponding decisions. Various estimation metrics are utilized towards robust and efficient risk management frameworks, with the most prevalent among them being the Value at Risk (VaR). VaR is a valuable risk-assessment approach, which offers traders, investors, and financial institutions information regarding risk estimations and potential investment insights. VaR has been adopted by the financial industry for decades, but the generated predictions lack efficiency in times of economic turmoil such as the 2008 global financial crisis and the COVID-19 pandemic, which in turn affects the respective decisions. To address this challenge, a variety of well-established variations of VaR models are exploited by the financial community, including data-driven and data analytics models. In this context, this paper introduces a probabilistic deep learning approach, leveraging time-series forecasting techniques with high potential of monitoring the risk of a given portfolio in a quite efficient way. The proposed approach has been evaluated and compared to the most prominent methods of VaR calculation, yielding promising results for VaR 99% for forex-based portfolios.

**Supplementary Information:**

The online version contains supplementary material available at 10.1007/s42521-022-00050-0.

## Introduction

Risk assessment in the financial sector has drawn considerable attention in recent years, as the extensive use of Value at Risk (VaR) among other risk management models proved to be inefficient in measuring the financial risk during recession periods, such as at the latest global financial crisis of 2008 and the 2020 COVID-19 pandemic. VaR, being originally designed for internal use in financial institutions, has become a key factor in determining capital requirements and conducting risk assessment, especially after the introduction of the Basel II Accord (De Waal et al. 2013). As a result of stricter regulations regarding capital management, alongside technological advances utilized in the financial sector, many institutions are actively exploring and promoting more accurate methods of monitoring and assessing the exposure to different risks (Elsinger et al. 2006; Zhao 2020).

Although VaR has caused high controversy throughout the years, it is considered to be a widely used internationally instituted financial risk model among several other financial tools and both quantitative analysts and regulatory authorities started paying more attention to VaR, since its standardization as a risk measure in 1996 by RiskMetrics (Longerstaey and Spencer 1996). The core of the criticism for VaR models derives from their simplistic assumptions and their insufficient results during periods of crises (Abad et al. 2014). The controversy raised over the VaR model can be summed in the indicative phrase of Einhorn and Brown in 2008, where VaR is compared to “an airbag that works all the time, except when you have a car accident” (Einhorn and Brown 2008).

The notion of VaR reflects the maximum expected loss of a portfolio over a given time horizon, at a predefined confidence level. Given the fact that VaR, besides its extensive use in the financial sector, has also been extensively studied in the academic literature, this paper does not further elaborate on the VaR definition. However, as several variants of VaR are emerging from the financial engineering and digital finance literature, we focus on the three major VaR categories, namely the non-parametric, the parametric, and the semi-parametric.

In the non-parametric models, assumptions regarding the distribution of the returns of the underlying portfolios are not required. The main advantage of this type of method is the low computational complexity. The Historical Simulation (HS) is the main representative of this category, where the empirical distribution of past portfolio returns is used to calculate VaR. The latter can be obtained by taking the required quantile of this distribution for a given history-window. The main disadvantage of this method is that it fails to capture unseen fluctuations that are not present in the utilized history-window (Chang et al. 2003).

Contrastingly, in parametric approaches, a valid model of the portfolio returns and their distribution should be theoretically defined prior to VaR estimation. On the one hand, most of the parametric models are simple in terms of implementation when Gaussian or Student-t distribution is assumed (Abad et al. 2014). On the other hand, this assumption does not hold for most of the financial time-series data. Some well-known methods falling in this category are the Variance–Covariance method (VC) and many GARCH-variants methods. For example, when VC method is applied assuming Gaussian distribution, a proper history-window should be defined, and the variance–covariance matrix of returns should be calculated, then the VaR can be obtained by Eq. 1

1 $$\begin{aligned} \text {VaR}^a = z_{1-a}\sqrt{W^{T}\sum {W}}, \end{aligned}$$

where *a*, *z*, and *w* are the confidence probability of VaR (i.e., 99%), the *z*-score, and the portfolio weights, respectively.

Finally, the semi-parametric VaR models are produced by combining the two aforementioned methods, where some assumptions are made either about the “error” distributions, its extremes, or the model dynamics. The Monte Carlo method (MC) is the main semi-parametric method, where scenarios are randomly generated for future returns of the portfolio, based on some non-linear pricing models. MC is more reliable comparing to historical and parametric approaches when dealing with complex portfolios and complicated risk factors. Its core assumption is that the risk factor has a known probability distribution, i.e., that market factors follow certain stochastic processes, which are used to estimate future returns (Abad et al. 2014).

Based on pertinent literature (Angelidis and Degiannakis 2018; Yamai and Yoshiba 2002), most of the existing VaR methods are facing various challenges. The most critical challenge is the severe VaR violations during which the portfolio realizes a loss exceeding the VaR value due to dependencies between the VaR predictions, especially for 99% confidence level. This is often the case when high market downturns occur. In addition, the high excess loss, beyond the VaR threshold that happens due to the fat tails of the financial time-series distribution and the leverage effect, is rarely taken into account. It should be also noted that despite the fact that there are several approaches providing a risk assessment management framework, the majority of related published literature examines single asset “portfolios” (i.e., S&P index). These drawbacks of the VaR methods reflect additional motivations for the approach presented in this paper.

The financial institutions started using VaR as a risk estimation metric to ensure their survival during catastrophic events, after the stock market crash on Wall Street in October 1987. The fact that the 2008 financial crisis resulted in an overall loss of $3.4 trillion among all major financial institutions over the world according to the International Monetary Fund (IMF) (Dattels and Miyajima 2009) is an example of the need of an efficient (innovative) VaR prediction methodology. Thus, it is crucial now that the COVID-19 pandemic has affected the global economy to a great extent (Das et al. 2021) to revise the risk-assessment tools and address their methodological limitations.

To this end, the research should be conducted in the context of portfolios consisted of certain type of financial assets (i.e., forex, bonds, and stocks). Given that VaR is independent of the type of assets comprising the portfolio and that there is abundance of open-source data for the majority of the forex (FX) instruments, portfolios based on FX assets were opted for.

This paper introduces a data-driven framework that predicts portfolios’ VaR, addressing the above-mentioned challenges with the following key innovations:

1. Integrates a continuous learning approach that considers the latest market prices avoiding clustered VaR violations and thus addressing the dynamic nature of financial data.
2. Is able to capture rare market events with very short training time by utilizing probabilistic forecasting based on auto-regressive recurrent neural networks in the context of VaR.
3. Goes beyond single-asset pre-trade/what-if analysis (i.e., asset-level) to portfolio pre-trade/what-if analysis (i.e., portfolio-level). It should be noted that this is achieved in (near) real time by eliminating the need to re-train the neural network model.

Even though considerable emphasis is given on portfolios composed of FX assets and daily VaR, the proposed approach is also applicable to other types of financial instruments and different time horizons, since it could be applied and optimized for various types of time-series. Moreover, the proposed framework has been evaluated with several loss functions and two different coverage tests.

This paper is structured as follows: Sect. 2 presents the related work in the relevant fields of study, while Sect. 3 describes the proposed methodological approach delivering details regarding the data and the evaluation scheme utilized. Sections 4 and 5 present the results of the back-testing to demonstrate and evaluate the performance and effectiveness of the proposed mechanisms. Finally, Sect. 6 closes the paper with recommendations on future research and further potentials of the current study.

## Related work

The foundations of VaR in finance as a risk-assessment approach were introduced in 1996 by Morgan and Reuters (Longerstaey and Spencer 1996). This parametric method became dominant in the financial sector and has been extensively utilized under the name “RiskMetrics model”, despite the fact it suffers from unrealistic assumptions such as the normally, independently, and identically distributed financial returns.

The aforementioned limitations, combined with the highly competitive and demanding nature of the financial markets, moved the research from the parametric method, to alternative independent directions. There are various studies comparing/back-testing different VaR approaches, with the one presented in Kuester et al. (2006) standing out. Summarizing its results, all the unconditional models produce clustered VaR violations, yet some may still pass as acceptable when considering only the (unconditional) violation frequencies. Though, this conclusion depends to some extent on the chosen window size, with less-parameterized models having an advantage as history-window size decreases from 1000 to 250. On the other hand, conditional VaR models lead to much more volatile VaR predictions and may arguably cause problems in allocating capital for trading purposes. Additionally, as discussed in Abad et al. (2014), most of the researchers focused on the family of GARCH models, arguing also that asymmetric GARCH models yield better results, but without statistically significant difference. Equally effective are many VaR applications, that utilize the Extreme Value Theory (EVT) approach. The key-characteristic of EVT is that it focuses on limiting the distribution of extreme returns observed over a long time-period, which is essentially independent of the distribution of the returns. There is an indicative comparative evaluation of the predictive performance of various VaR models, emphasizing on the two EVT-based methodologies, Peak Over Threshold model (POT) (Novak 2011), and block maxima model (BM) (Mcneil 1998). Its results demonstrated that, although some traditional methods might yield comparable results at the conventional confidence levels, the EVT methodology produces the most accurate forecasts of extreme losses for high confidence levels (Bekiros and Georgoutsos 2005).

Despite the widespread use of various econometric models regarding VaR estimation and modeling of financial returns, the rise of machine learning and deep learning models offered an improved toolkit to financial firms, introducing innovative and more effective approaches and automating many financial tasks. In the last few years, several studies have been carried out, such as (Lim and Zohren 2020; Sen et al. 2019), analyzing how some of the most prominent deep learning architectures [such as Recurrent Neural Networks (RNNs) and Long Short-Term Memory (LSTM) (Gers et al. 2000)] can be used for time-series forecasting. Such models have been widely applied across different domains, due to their ability to model non-linear temporal patterns. For instance, in Neuneier (1996) and Xiong et al. (2018), a neural network-based reinforcement learning model is used to perform portfolio management. In Weng et al. (2017), an ensemble model of machine learning algorithms has been utilized to predict stock market movement, proving that an LSTM neural network has a high potential for predicting financial time-series. Moreover, a Generative Adversarial Networks (GAN)-based model (Goodfellow et al. 2014) has been proposed to generate synthetic representative financial data sets, demonstrating that the synthetic distributions share similar characteristics with the real data (Pfenninger et al. 2021). Finally, a combination of wavelet analysis and an LSTM neural network enables to capture the complex features of financial time-series, such as non-linearity, non-stationarity, and sequence correlation (Yan and Ouyang 2018).

As far as the VaR estimation is concerned, two key challenges arise from the relative literature: (i) The first one refers to the excess loss of VaR violations, which is rarely taken into account. For instance, in Hendricks (1996), the authors argued that, although a 99% risk measure may sound as if it is capturing essentially all of the relevant events, the other 1% of events can in extreme cases entail losses substantially in excess of the risk measures generated on a daily basis. (ii) The second challenge is that most of VaR 99% models produce more violations than the nominal confidence probability, with limited back-testing regarding the coverage and independence of VaR estimations. Moreover, most of the related research papers focus on certain financial indexes (i.e., S&P) as a univariate time-series, without taking into account the correlations among the assets that comprise the examined portfolio.

Based on the above, the main contribution of the current research is twofold. First, a novel probabilistic approach for VaR prediction is proposed based on DeepAR, providing the full predictive distribution and allowing decision-makers to optimize their actions. This approach is performing better in VaR 99% than the most prevalent VaR estimation methods. Second, evaluation/back-testing procedures based not only on univariate assets but also on portfolios are presented. Furthermore, various evaluation metrics and statistical tests were utilized to illustrate the efficiency of the proposed approach.

## Proposed approach

An innovative framework for portfolio VaR estimation is proposed, utilizing probabilistic deep neural networks (Mohebali et al. 2020). However, regularized datasets are not available, as hardly any hedge fund or individual trader is willing to disclose proprietary information of their portfolios, while in most of the relevant studies only a univariate index (like S&P) is leveraged as a portfolio. In our approach, the VaR of each asset ($\text {VaR}_i$) is initially calculated, then the portfolio’s VaR ($\text {VaR}_p$) is derived from the combination of $\text {VaR}_i$, taking into account their correlations and their corresponding weights. Several random portfolios have been created to evaluate our results for different cases and scenarios.

Furthermore, to evaluate the proposed model, five different VaR methods were explored, namely, GARCH, RiskMetrics (RM), HS, BiGAN, and MC. The evaluation was based on several loss metrics, such as the number of VaR violations, quadratic loss and firm loss, and coverage tests described in Sect. 3.2.3.

In our approach, both VaR and Profit and Loss (PnL) are expressed in log-return terms, while the terms PnL and portfolio returns *r* are used interchangeably in the remainder of this paper. Mathematically, let $\text {PnL}_t = r_t = \log {\frac{P_t}{P_{t-1}}}$ be the log-returns, and $P_t$ is the close price of the financial instrument on day *t*. The 1-day VaR on day *t* is defined as

2 $$\begin{aligned} P( r_t \le \text {VaR}^{\alpha }_{t})= 1 - \alpha . \end{aligned}$$

As shown in Eq. 2, VaR is expressed in return terms, thus given a distribution of return, VaR can then be determined and expressed in terms of a percentile of the return distribution (Christoffersen et al. 2001). Specifically, if $q_{\alpha }$ is the $\alpha$-th percentile of the continuously compounded return, then VaR can be expressed as

3 $$\begin{aligned} \text {VaR}^a = q_{1-a}. \end{aligned}$$

### DeepVaR

The approach presented in this paper exploits deep neural networks and probabilistic forecasting. Having tested different probabilistic time-series forecasting models, DeepAR Estimator as described in Salinas et al. (2020) has been selected as the core of the proposed framework, the so-called DeepVaR.

DeepAR is based on an auto-regressive recurrent neural network model, specifically designed for multivariate time-series modeling producing accurate probabilistic forecasts. It models the conditional distribution $P(z_{i, t_0: T} \mid z_{i, 1: t_0 - 1})$ of the time-series $z_i$ for future time-steps from $t_0$ to *T* given the past values of the $z_i$ from time-step 1 to $t_{0} - 1$. It is assumed that the model distribution $Q_{\theta }(z_{i, t_0: T} \mid z_{i, 1: t_0 - 1})$ consists of a product of likelihood factors. The latter are maximized to train a deep neural network (RNN) that learns the distributions’ parameters. Several samples can be easily generated from the estimated distribution in an MC fashion. Moreover, this algorithm can be trained with several similar time-series simultaneously, enabling cross-learning between them.

This algorithm has been applied successfully in several business sectors and real-life scenarios, such as food safety (Makridis et al. 2020), where a set of probabilistic techniques were introduced to provide insights regarding potential food recalls, in retail sector predicting the number of sales per product (Khan et al. 2020) and in industry estimating electricity demand (Wu et al. 2020). This innovative algorithm has been complemented with additional features as part of the overall framework presented in this paper. These features (such as the portfolio-level predictions and the continuous learning) were deemed essential to successfully utilize DeepVaR in the finance sector. The framework predicts VaR of FX portfolios, where the individual time-series share similar dependencies and the overall goal is to draw the portfolio’s returns distribution.

Furthermore, DeepVaR proposes a continuous learning approach instead of a classical machine learning pipeline where the model is trained once and for several hours in a large dataset. Contrastingly, in DeepVaR, the model is retrained on the latest market data, addressing the dynamic nature of financial data while avoiding at the same time model bias and drift, serial correlation between VaR estimations and clustered VaR violations (Mehrabi et al. 2021). To this end, DeepAR parameters were optimized to enable model training in a timely manner ($<13$ s), making it applicable even for intra-day VaR estimations.

**Fig. 1:**
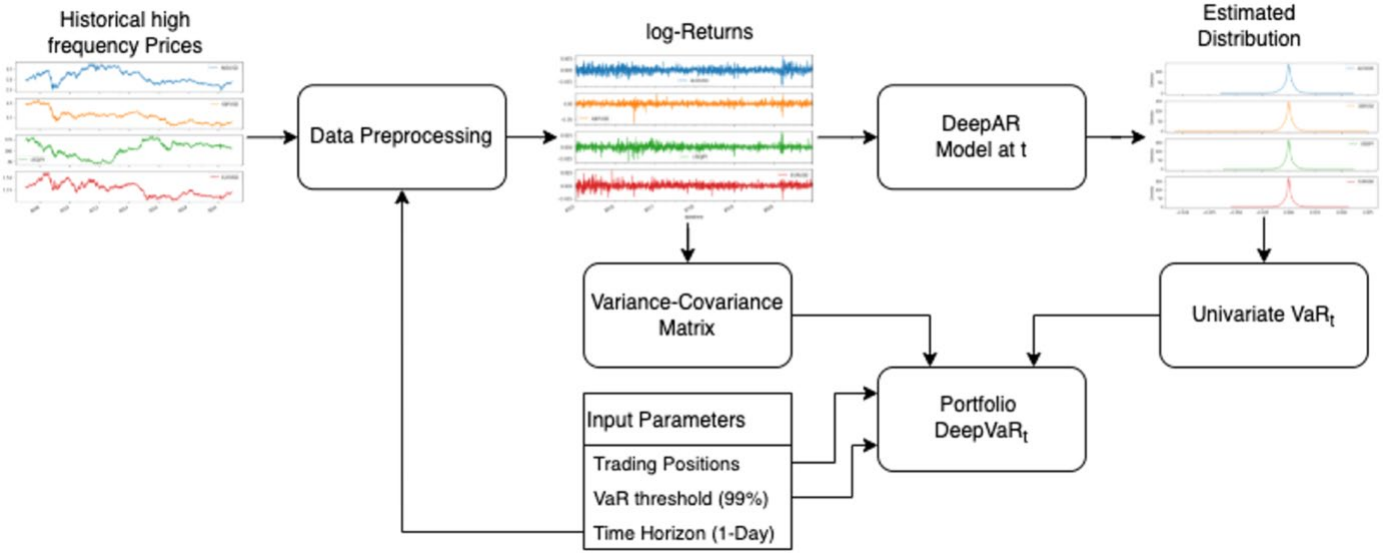
Conceptual architecture of DeepVaR framework

As depicted in Fig. 1 and further analyzed in terms of sequence flows by Algorithm 1, the DeepVaR framework performs estimations on asset-level (i.e., for a single VaR) at each time-step *t*. Historical market prices $x_i$ from $t_0$ to $t-1$ of multiple instruments *i* are ingested into the framework simultaneously. During the data preprocessing step, the input data are initially resampled to match the frequency of the selected VaR time horizon and then are transformed to log-returns ($r_{i,t}$). For example, in the case of minute data and daily VaR selection, the input time-series is transformed to daily log-returns. The latter is used to train the DeepAR model and to estimate the distribution of each time-series (i.e., asset-level) for time-step *t*. With the distribution of the assets’ returns available, $\text {VaR}_{i,t}$ can be obtained from Eq. 3. In the last step, portfolio-level predictions, i.e., portfolio $\text {VaR}_{p,t}$ is estimated based on the returns variance–covariance matrix, $\text {VaR}_{i,t}$ and the input weight on each asset (see Sect. 5 for more details). It is also noted that the process of calculating $\text {VaR}_p$ from $\text {VaR}_{i}$ requires only matrices multiplications (see Eq. 15). Thus, no training is required and, therefore, the overall process is quite time-efficient. Thus, DeepVaR could be also used for what-if analysis comparing portfolios’ risk against the different weights on input assets/instruments.

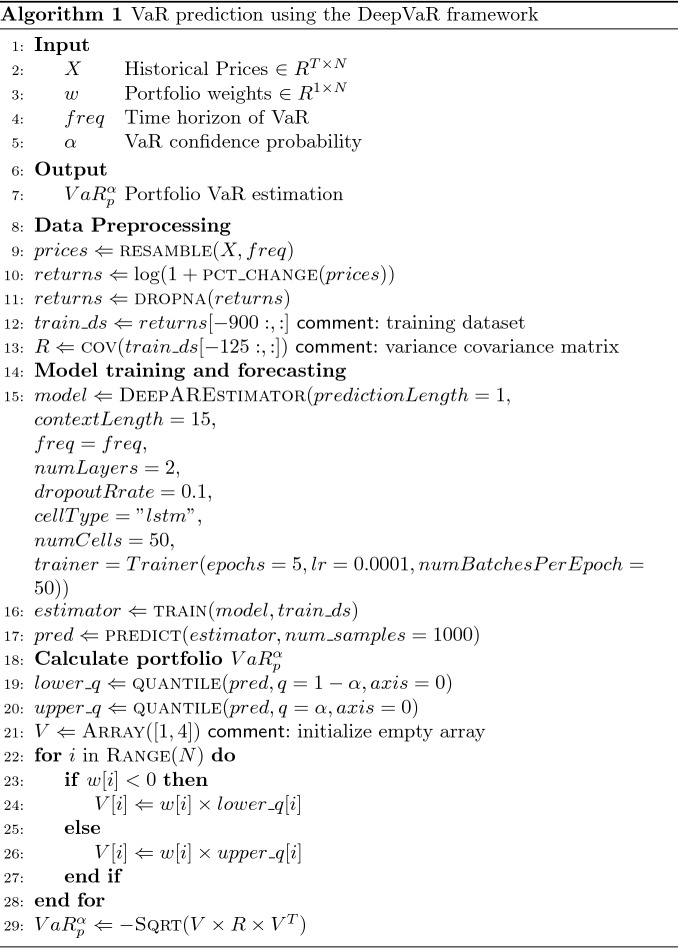



Additionally, due to noisy data, the initialization of the weights of the RNN model, which is random due to the stochastic nature of this optimization process, had quite an impact on the performance. This pitfall is common when noisy data are concerned as multiple local minima in the loss surface exist. This is quite challenging as the loss surfaces are generally non-convex and may have multiple saddle points making it difficult to achieve model stability. To tackle this challenge, different models (10 in total) with different random seeds (i.e., initial weights) were trained and ensembled as a Bagging model, as proposed by Montavon et al. (2012). All these models share the same hyper-parameters such as 900 observations training set, 5 epochs, 1-day prediction length, and 15-day context length, and were fine-tuned using grid-search technique and intuition, as a fundamental step of the machine learning pipeline. Moreover, the selected model features the ADAM optimizer (Kingma and Ba 2014) with the learning rate set to 0.0001, 2 LSTM layers with 50 cells each, and dropout 10%.

### Evaluation approach

In this section, a brief description of the employed baseline models is initially given, and then, the utilized data along with the portfolio contraction procedure are analyzed. Considerable emphasis is also given on both the evaluation metrics and the statistical tests that enable the comparison between several VaR models.

#### Baseline techniques

Towards evaluating the proposed DeepVaR framework, a comparison against the four most used VaR estimation techniques and one deep learning model was performed. A short description of these techniques follows.

GARCH type models have been widely used for VaR estimation, producing considerably well results in exchange rate data (So and Philip 2006). Such models can capture the time-varying volatility feature characterizing financial time-series. Thus, a GARCH model can be utilized to predict the future volatility of the returns. Since GARCH(1,1) model was found to be adequate to many financial time-series (Bollerslev et al. 1992), we have chosen it as one of the three baseline models, which can be described as

4 $$\begin{aligned} r_t= & {} \mu + \epsilon _t, \end{aligned}$$

5 $$\begin{aligned} \epsilon _t= & {} \sigma _te_t, e_t\sim N(0,1), \end{aligned}$$

6 $$\begin{aligned} \sigma _t^2= & {} \omega +\alpha \epsilon ^2_{t-1}+\beta \sigma ^2_{t-1}. \end{aligned}$$

Future returns $r_{t+1}$ can be obtained by estimation of the parameters $\omega ,\alpha , \beta$ in Eq. 6 using the maximum-likelihood method. To have comparable results to the DeepVaR, the parameters of GARCH(1, 1) model are estimated over a sample of 900 observations.

The second baseline technique refers to the RM model for VaR estimation. As proposed in Longerstaey and Spencer (1996), it is a GARCH(1,1) variant, modeling the volatility of returns at the next time-step as exponential weighted moving average of the past volatilities $\sigma ^2_{t} = \lambda \sigma ^2_{t-1\mid t-2} + r^2_{t-1}(1-\lambda ), \lambda = 0.94$, while the mean of returns $\mu$ considered is equal to zero. The value of the decay factor $\lambda$ implies that the effective historical data to forecast future volatility are approximately 74 days.

The third baseline technique, HS, uses a configurable number of past historical observations to calculate the portfolio’s the actual percentiles of this observation period as value-at-risk measures. For example, for an observation period of 1000 days, the 99th percentile HS value-at-risk measure is the 11th largest loss observed in the sample of 1000 outcomes (because the 1 percent of the sample that should exceed the risk measure equates to 10 losses) (Hendricks 1996). The effective historical window for the HS model was fine-tuned to 1000 days.

In addition, an MC-based VaR model has been employed to evaluate the performance of the proposed solution, which also includes an MC procedure. The MC model produces random samples from the normal distribution to estimate the future distribution of the portfolio returns. The generated distribution is then used to calculate VaR. The input parameters (i.e., $\mu , \sigma ^2$) for the MC model have been calculated from the last 900 historical data that offered better performance in comparison to a lower number of historical observations.

Another baseline technique baseline technique leverages Bidirectional Generative Adversarial Networks (BiGAN) (Donahue et al. 2016) towards modeling the joint probability distribution of the portfolio returns without the need to specify their distribution explicitly. The BiGAN consists of three neural networks, a generator (G) that learns to produce realistic synthetic samples from the latent space *z*, an encoder (E) which learns to map data *x* to latent representations *z*, and a discriminator (D) distinguishing jointly real from synthetic samples and real from synthetic encodings. After the BiGAN training, the generator is used to produce samples that will be close to the actual returns’ distribution, and based on them, VaR is obtained. The utilized BiGAN, based on the code available in GitHub,^1^ was evaluated following the continuous learning approach (i.e., retraining the model at each new data observation) introduced by the DeepVaR framework. To have comparable results to the DeepVaR, the selected training data size was set to 900 data points, while 75 epochs were required to calibrate the binary cross-entropy loss of BiGAN components.

#### Dataset description

In the context of the evaluation of the proposed approach against the baseline techniques, the four FX instruments (AUDUSD, GBPUSD, USDJPY, and EURUSD) with the highest liquidity among the rest have been chosen as the underlying dataset. Specifically, the dataset consists of daily close prices, ranging from 2007/01/01 to 2020/12/18, with the data being obtained from http://www.eatradingacademy.com. The daily prices, represented as time-series, were transformed to log-returns to make them stationary, while the VaR predictions are measured in this scale.

**Fig. 2:**
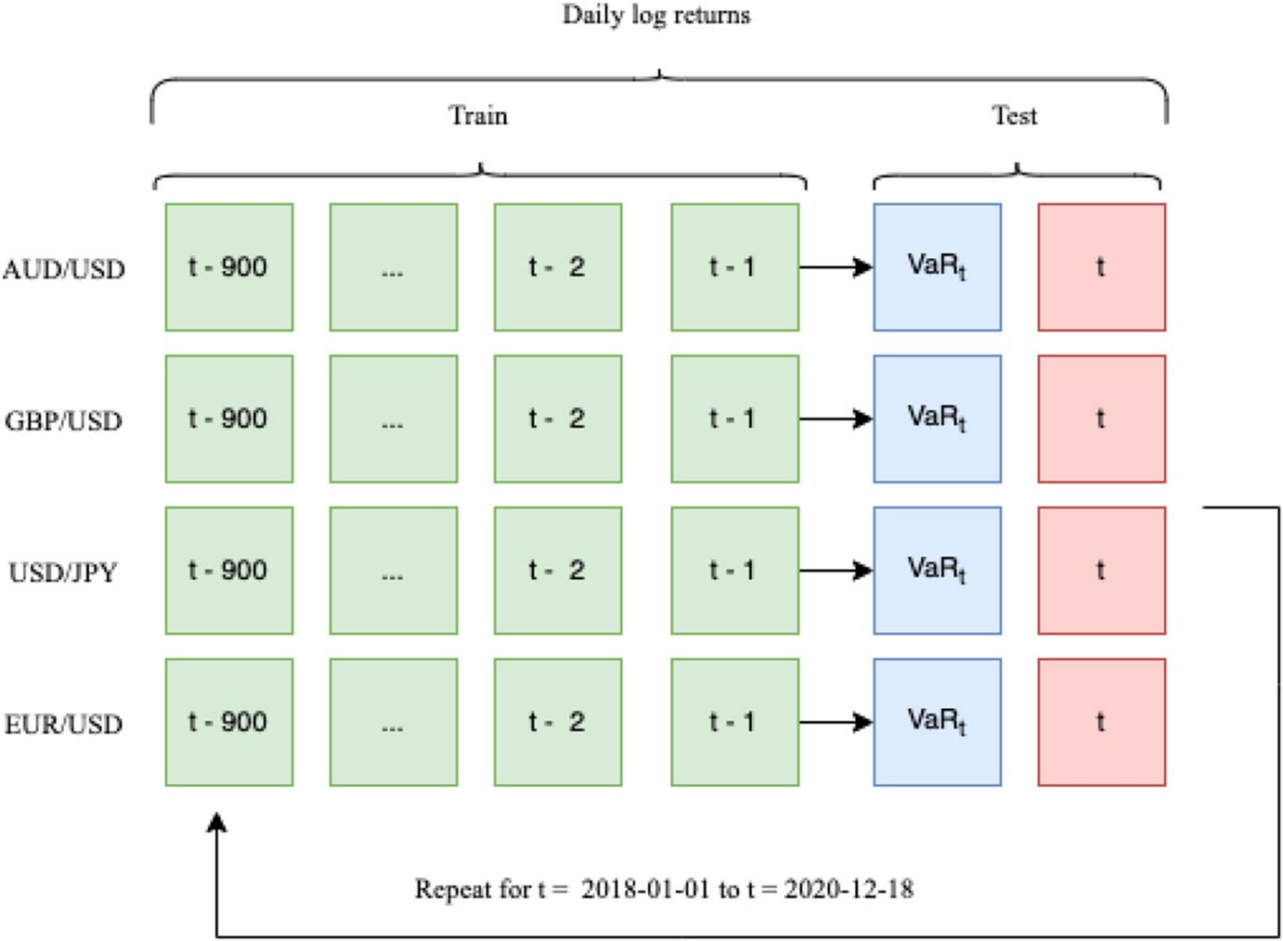
Training dataset for rolling window VaR estimation

First, the 1-day VaR of each FX pair is calculated from 2018/01/01 to 2020/12/18 in a rolling window prediction format with this time period serving also as test dataset for the models’ evaluation. The training window, for the DeepVaR, BiGAN, and GARCH models, was set to 900 data points consisting of the latest 900 daily log-returns of each FX asset (see also Fig. 2). For the HS model, the historic window was fine-tuned to 900 days, contrary to RiskMetrics, where only the last 74 days were taken into account.

Second, to increase the reliability of our results, 1000 portfolios have been created reflecting the historical behavior of 1000 different traders. The asset allocation in each of these portfolios was generated randomly and expressed as different proportions of the four FX instruments. These portfolios may include both long and short positions (i.e., positive and negative weighting coefficients), while the sum of the absolute weights is equal to one. It should be mentioned that limitations derived from Modern Portfolio Theory (Francis and Kim 2013) regarding the generation of the portfolios are out of the scope of this research as they do not affect its findings.

Finally, both the $VaR_p$ baseline models and the proposed approach were evaluated for the period from 2018-01-01 to 2020-12-18, applying an 1-day rolling window forecast. To this end, the previous 125 days were used to calculate the correlation between the $VaR_i$’s, interpolating the latter to a $VaR_p$.

#### Evaluation metrics

This section describes the evaluation metrics utilized to assess the proposed DeepVaR approach. These metrics are key towards interpreting the outcomes of the back-testing, as each one highlights different aspects of the results.

To begin with, a common variable in most of evaluation metrics is the *Hit Variable* associated with the ex-post observation of a VaR exception at time *t* is denoted as $I_t$ where

7 $$\begin{aligned} I_t = {\left\{ \begin{array}{ll}1,&{} \text {if } \text {VaR}_t> \text {PnL}_t\\ 0,&{} \text {otherwise}.\end{array}\right. } \end{aligned}$$

Expected violations: are the maximum number of allowed exceedances (i.e. $\text {VaR}>\text {PnL}$). It is defined by the confidence probability $\alpha$ of VaR and the number of the days in the examined period.

The Expected Violations (*E*[*v*]) of $\text {VaR}_{\alpha }$ are

8 $$\begin{aligned} E[v] = (1-\alpha ) N_{\text {days}}. \end{aligned}$$

Number of violations: the number of the VaR violations is denoted as $N_{\text {violations}}$.

9 $$\begin{aligned} N_{\text {violations}} = \sum ^T_{t=1}I_t. \end{aligned}$$

Violation rate: the ratio of the Number of Violations over the examined period. This number should be less than the $1-\alpha$ to have a $\text {VaR}^a$ model with good coverage.

10 $$\begin{aligned} r_{\text {Violations}}=\frac{N_{\text {Violations}}}{N_{\text {days}}}. \end{aligned}$$

Quadratic Loss ($l_{\text {QL}}$): takes into account the quadratic magnitude of the exceedances

11 $$\begin{aligned} l_{\text {QL}} = \sum ^N_{t=1}I_t(1+(\text {PnL}_{t}-\text {VaR}_{t})^2). \end{aligned}$$

Smooth loss ($l_Q$) (González-Rivera et al. 2004): is a loss function that penalizes more heavily with weight (1–a) the observations for which $\text {PnL} - \text {VaR} < 0$. Smaller $l_Q$ indicates a better goodness of fit

12 $$\begin{aligned} l_Q = \frac{1}{N}\sum ^N_{t=1}(\alpha -(1+e^{dm})^{-1})m, \end{aligned}$$

where $$d=25$$, $$m=\text {PnL}_t-\text {VaR}^{\alpha }_t$$.

Tick loss ($l_{T}$): this loss penalizes exceedances with weight $\alpha$ and non-exceedances with weight $1-\alpha$, meaning that more conservative VaR estimations are producing higher Tick Loss

13 $$\begin{aligned} l_{T} = \sum ^N_{t=1}(\alpha - I_t)(\text {PnL}_{t}-\text {VaR}^{\alpha }_{t}). \end{aligned}$$

Firm loss ($l_F$) (Sarma et al. 2003): VaR is generally used by firms for internal risk management. However, there is a trade-off between the risk minimization and the profit maximization. A VaR estimator which reported ‘too high’ values of VaR would force the firm to hold ‘too much’ capital, imposing the opportunity cost of capital upon the firm

14 $$\begin{aligned} l_F = {\left\{ \begin{array}{ll}(\text {PnL}_{t}-\text {VaR}_{t})^2,&{} \text {if } \text {PnL}_t<\text {VaR}_t \\ -a \text {VaR}_t,&{} \text {otherwise},\end{array}\right. } \end{aligned}$$

where *a* measures the opportunity cost of capital and it was set equal to 1.

Additionally, according to Christoffersen (1998), VaR forecasts are valid if and only if the violation process $I_t$ satisfies the following two assumptions: (i) The unconditional coverage (UC) hypothesis: the unconditional probability of a violation must be equal to the $\alpha$ coverage rate. (ii) The independence (IND) hypothesis: VaR violations observed at two different dates must be independently distributed

In the frame of our research, validity of these assumptions is tested by exploiting both the Christoffersen conditional coverage test and the Dynamic Quantile (DQ) test proposed by Engle and Manganelli (2004). The former jointly examines whether the percentage of exceptions is statistically equal to the one expected and whether VaR violations are serially independent. This is achieved by an independence test, which aims to reject VaR models with clustered violations. The likelihood ratio statistic of the conditional coverage test is $\text {LR}_{\text {cc}} = \text {LR}_{\text {uc}} + \text {LR}_{\text {ind}}$, which is asymptotically distributed $X^2$, and the $\text {LR}_{\text {ind}}$ statistic is the likelihood ratio statistic for the hypothesis of serial independence against the first-order Markov dependence. The latter examines whether the exception indicator $I_t$ is uncorrelated with any variable that belongs to the information set $\Omega _{t-1}$ available when the VaR was calculated.

The main argument (hypothesis) of this work is that an RNN-based model is able to predict the future returns of the input time-series more accurately than well-established econometric models. The second argument lies in the fact that the utilized DeepAR model is fed with all the input time-series simultaneously, enabling cross-learning between them. As a result, changes in the dynamics of the one time-series may affect the predicted distributions of the other time-series.

The evaluation of the examined models in the following section proves that: (i) DeepVaR is able to capture (i.e., predict) the abrupt changes of the input time-series, and (ii) DeepVaR is the only model that avoids clustered VaR violations due to its non-linear nature, which also allows the proposed model to report uncorrelated VaR estimations.

#### Experiment details

GluonTS Alexandrov et al. (2019), Tensorflow Abadi et al. (2015), and arch Sheppard (2020) python libraries were utilized for the development of DeepVaR, BiGAN, and GARCH models, respectively, with NumPy (Harris et al. 2020) opted for the rest baseline models. Experiments are run on a desktop computer with an AMD Ryzen 5 5600x 6-Core CPU, 32GiB of RAM, and an NVIDIA GeForce RTX 3070 GPU. However, neural network training is performed in the CPU, and thus, further improvement in DeepVaR training time is feasible. Table 1 summarizes the required mean time in seconds per model to obtain the quantiles (e.g., $q_1, q_{99}$) needed for the VaR estimation of both single and four assets portfolios. The fourth column of the table indicates the relative difference in calculation time between the two different input sizes. According to these findings, it is obvious that despite the fact that deep learning-based models require significantly more time to estimate VaR than the other models, that time ($\approx 12.5s$) is very low, enabling VaR estimation even for intra-day trading applications. Moreover, the input size has a minimal effect on deep learning models’ training time which leverage matrices operations to parallelize computations. In contrast, estimation time in econometric models such as GARCH is linearly dependent on the number of the input time-series.

**Table 1:** Mean running time to estimate VaR quantiles

Model	1 Asset (s)	4 Assets (s)	Rel. Difference (%)
DeepAR	12.457834	12.533903	0.61
HS	0.000201	0.000398	98.01
RM	0.003418	0.013461	293.83
GARCH	0.009435	0.038732	310.51
BiGAN	20.467264	20.947305	2.35
MC	0.000567	0.002108	271.78

## Univariate VaR performance

**Fig. 3:**
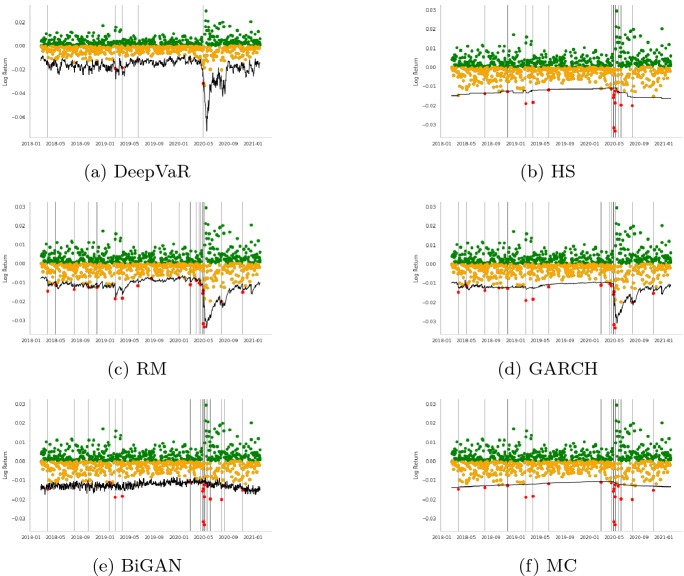
AUDUSD: $VaR^{99\%}$ performance per model. In each figure, the VaR estimation of each model (black line) is depicted against the true PnL (green and yellow dots, for positive and negative returns, respectively). The red dots represent the VaR violations

**Fig. 4:**
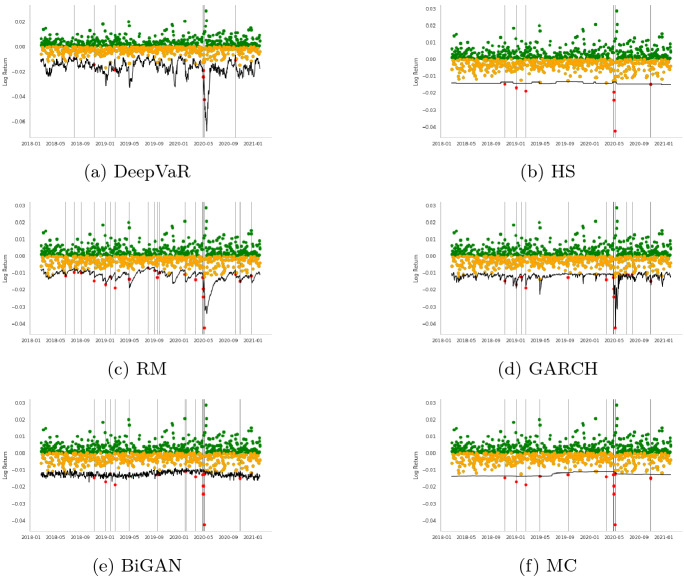
GBPUSD: VaR$^{99\%}$ performance per model. In each figure, the VaR estimation of each model (black line) is depicted against the true PnL (green and yellow dots, for positive and negative returns, respectively). The red dots represent the VaR violations

**Fig. 5:**
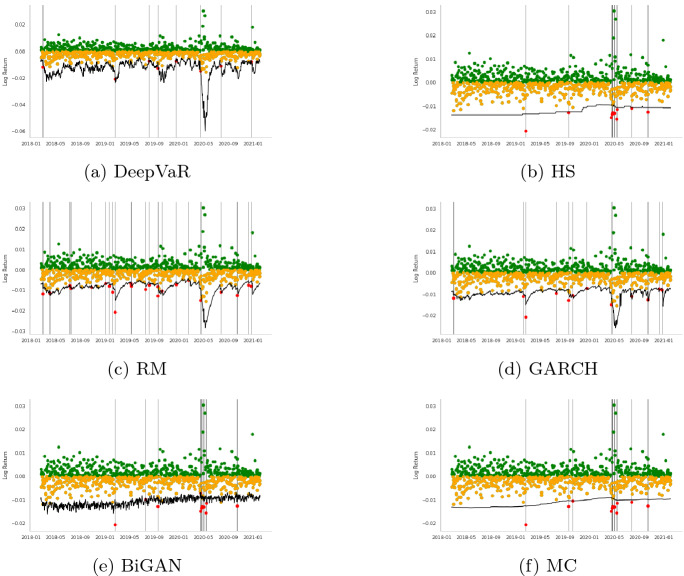
USDJPY: VaR$^{99\%}$ performance per model. In each figure, the VaR estimation of each model (black line) is depicted against the true PnL (green and yellow dots, for positive and negative returns, respectively). The red dots represent the VaR violations

**Fig. 6:**
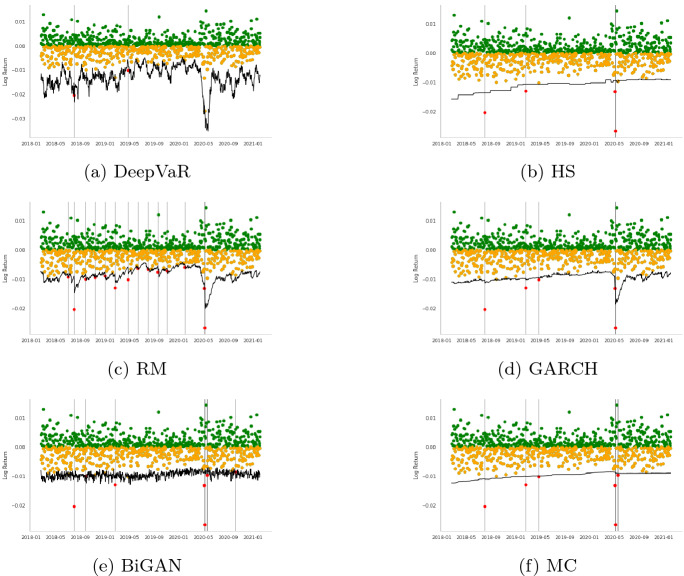
EURUSD: VaR$^{99\%}$ performance per model. In each figure, the VaR estimation of each model (black line) is depicted against the true PnL (green and yellow dots, for positive and negative returns respectively). The red dots represent the VaR violations

In this section, initially, we present the VaR estimations for each FX asset separately for the period 2018-01-01 to 2020-12-18, following the dataset descriptions Sect. 3.2.2. Tables 2, 3, 4, 5, 6, 7, 8, 9 and Figs. 3, 4, 5, 6 summarize the performance of each model. In each figure, the VaR estimation of each model (black line) is depicted against the true portfolio returns (green and yellow dots, for positive and negative returns, respectively). The red dots are the VaR violations. The complementary tables following each model’s presentation contain the aforementioned evaluation metrics and statistical tests.

The first time-series under consideration is the AUDUSD currency pair which is characterized by very strong liquidity due to the large amount of Australian exports. As depicted in Table 2, the DeepVaR model outperforms the rest of the models in this time-series, having the lowest loss over all the examined loss metrics. Additionally, according to Table 3, which presents the results of Christoffersen and DQ tests for 99% VaR estimation, all the models, except DeepVaR, have been rejected for not having the “correct unconditional coverage”. These outcomes can also be seen in Fig. 3. The advantage of the DeepVaR model emerges from the fact that it adapts to stricter VaR estimates as AUDUSD volatility increases, while classic VaR models suffer from clustered VaR violations (e.g., at the end of May 2021).

**Table 2:** Performance of VaR$^{99\%}$ models in AUDUSD series

Model	*E*[*v*]	*v*	$r_v$	$l_{QL}$	$l_Q$	$l_T$	$l_F$
DeepVaR	9.28	**5**	**0**.**00539**	**0**.**00539**	$-$ **0**.**00632**	**0**.**00020**	**0**.**02334**
HS	9.28	15	0.01616	0.01617	$-$0.00519	0.00022	0.02923
RM	9.28	21	0.02263	0.02263	$-$0.00472	0.00021	0.03457
GARCH	9.28	17	0.01832	0.01832	$-$0.00488	**0**.**00020**	0.03059
BiGAN	9.28	20	0.02155	0.02155	$-$0.00502	0.00023	0.03403
MC	9.28	18	0.01940	0.01940	$-$0.00489	0.00022	0.03149

Values in bold indicate the model(s) with the best performance per evaluation metric (column)

**Table 3:** Coverage and independence tests of VaR$^{99\%}$ models in AUDUSD series

Model	$\text {LR}_{\text {uc}}$	$\text {LR}_{\text {ind}}$	$\text {LR}_{\text {cc}}$	DQ
DeepVaR	2.386 [0.122]	0.054 [0.816]	2.441 [0.295]	2.158 [0.905]
HS	3.014 [0.083]	$$10.682^{**}$$ [0.001]	$$13.696^{**}$$ [0.001]	$$124.125^{**}$$ [0.0]
RM	$$11.036^{**}$$ [0.001]	2.931 [0.087]	$$13.966^{**}$$ [0.001]	$$31.907^{**}$$ [0.0]
GARCH	$$5.224^{*}$$ [0.022]	1.013 [0.314]	$$6.237^{*}$$ [0.044]	$$17.616^{**}$$ [0.007]
BiGAN	$$9.424^{**}$$ [0.002]	$$7.211^{**}$$ [0.007]	$$16.635^{**}$$ [0.0]	$$106.141^{**}$$ [0.0]
MC	$$6.512^{*}$$ [0.011]	$$8.445^{**}$$ [0.004]	$$14.957^{**}$$ [0.001]	$$108.176^{**}$$ [0.0]

The *p* values are in brackets

The next examined FX instrument is GBPUSD associated with two of the largest western economies with very strong trading relationships. According to Table 4, the DeepVaR model has the lowest number of violations, quadratic, smooth, and tick loss, while the GARCH model shares similar performance to DeepVaR in terms of tick loss, with the HS model illustrating the lowest firm loss. Figure 4 indicates that DeepVaR and HS fail to capture the negative returns almost at the same dates, while the other two models have more frequent VaR violations. Results in Table 5 denote that all the models, except from the RiskMetrics model, passed the Christoffersen’s test; however, all the examined models failed in the DQ test. Thus, the VaR violations of each model in GBPUSD time-series are serially dependent, although Fig. 4 shows that this issue is more pronounced in the RM and GARCH models.

**Table 4:** Performance of VaR$^{99\%}$ models in GBPUSD series

Model	*E*[*v*]	*v*	$r_v$	$l_{\text {QL}}$	$l_Q$	$l_T$	$l_F$
DeepVaR	9.28	**7**	**0**.**00754**	**0**.**00754**	$-$ **0**.**00617**	**0**.**00019**	0.02454
HS	9.28	8	0.00862	0.00862	$-$0.00557	0.00020	**0**.**02280**
RM	9.28	22	0.02371	0.02371	$-$0.00467	0.00021	0.03530
GARCH	9.28	14	0.01509	0.01509	$-$0.00485	**0**.**00019**	0.02710
BiGAN	9.28	16	0.01724	0.01724	$-$0.00502	0.00022	0.02969
MC	9.28	13	0.01401	0.01401	$-$0.00508	0.00021	0.02663

Values in bold indicate the model(s) with the best performance per evaluation metric (column)

**Table 5:** Coverage and independence tests of VaR$^{99\%}$ models in GBPUSD series

Model	$\text {LR}_{\text {uc}}$	$\text {LR}_{\text {ind}}$	$\text {LR}_{\text {cc}}$	DQ
DeepVaR	0.613 [0.434]	$$4.258^*$$ [0.039]	4.871 [0.088]	$$27.815^{**}$$ [0.0]
HS	0.184 [0.668]	3.713 [0.054]	3.897 [0.142]	$$31.297^{**}$$ [0.0]
RM	$$12.745^{**}$$ [0.0]	0.366 [0.545]	$$13.11^{**}$$ [0.001]	$$36.725^{**}$$ [0.0]
GARCH	2.108 [0.147]	1.623 [0.203]	3.731 [0.155]	$$35.0^{**}$$ [0.0]
BiGAN	$$4.055^{*}$$ [0.044]	$$4.868^{*}$$ [0.027]	$$8.923^{*}$$ [0.012]	$$98.703^{**}$$ [0.0]
MC	1.347 [0.246]	$$6.491^{*}$$ [0.011]	$$7.838^{*}$$ [0.02]	$$77.377^{**}$$ [0.0]

The *p* values are in brackets

VaR is also estimated for the US Dollar to Japanese Yen currency pair (USDJPY) which is the second most commonly traded pair after EURUSD. Generally, USDJPY has very high liquidity; however, JPY can also be viewed as a ‘safe haven’ currency during periods of global economic uncertainty. As shown in Table 6, DeepVaR model has the best overall performance, besides tick loss where the GARCH model performs better. As far as the coverage and independence tests illustrated in Table 7, both the DeepVaR and the GARCH models showcase promising results. However, only DeepVaR has less VaR violations than the nominal threshold. It is also noted that DeepVaR reports stricter VaR estimates than the rest of the models during the high volatility period in May 2020, as shown in Fig. 5. This is due to the shared dependencies between the four examined time-series during the training of the DeepAR algorithm.

**Table 6:** Performance of VaR$^{99\%}$ models in USDJPY series

Model	*E*[*v*]	*v*	$r_v$	$l_{\text {QL}}$	$l_Q$	$l_T$	$l_F$
DeepVaR	9.28	**8**	**0**.**00862**	**0**.**00862**	$-$ **0**.**00493**	0.00015	**0**.**02146**
HS	9.28	11	0.01185	0.01185	$-$0.00489	0.00016	0.02387
RM	9.28	24	0.02586	0.02586	$-$0.00366	0.00015	0.03456
GARCH	9.28	13	0.01401	0.01401	$-$0.00396	**0**.**00013**	0.02351
BiGAN	9.28	12	0.01293	0.01293	$-$0.00439	0.00016	0.02354
MC	9.28	12	0.01293	0.01293	$-$0.00460	0.00016	0.02411

Values in bold indicate the model(s) with the best performance per evaluation metric (column)

**Table 7:** Coverage and independence tests of VaR$^{99\%}$ models in USDJPY series

Model	$\text {LR}_{\text {uc}}$	$\text {LR}_{\text {ind}}$	$\text {LR}_{\text {cc}}$	DQ
DeepVaR	0.184 [0.668]	0.139 [0.709]	0.324 [0.851]	2.857 [0.827]
HS	0.308 [0.579]	2.477 [0.116]	2.785 [0.248]	$$49.276^{**}$$ [0.0]
RM	$$16.439^{**}$$ [0.0]	0.207 [0.649]	$$16.646^{**}$$ [0.0]	$$36.755^{**}$$ [0.0]
GARCH	1.347 [0.246]	0.37 [0.543]	1.717 [0.424]	8.448 [0.207]
BiGAN	0.743 [0.389]	$$7.143^{**}$$ [0.008]	$$7.886^*$$ [0.019]	$$81.484^{**}$$ [0.0]
MC	0.743 [0.389]	2.159 [0.142]	2.902 [0.234]	$$45.667^{**}$$ [0.0]

The *p* values are in brackets

The last utilized currency pair, EURUSD, is the most widely traded forex pair in the market as it comprises the currencies of two of the world’s biggest economies. The results of the various loss metrics are summarized in Table 8. DeepVaR presents the lowest quadratic and smooth loss, as well as the fewest VaR violations. GARCH model performed better than the other models in terms of tick and firm loss. In addition, Table 9 shows that all models passed Christoffersen coverage and independence test, but only DeepVaR and RM succeeded in the DQ test. Finally, Fig. 6 shows that DeepVaR is the only model that did not suffer any VaR violation during the EURUSD volatility shift in May 2020.

**Table 8:** Performance of VaR$^{99\%}$ models in EURUSD series

Model	*E*[*v*]	*v*	$r_v$	$l_{\text {QL}}$	$l_Q$	$l_T$	$l_F$
DeepVaR	9.28	**2**	**0**.**00216**	**0**.**00216**	$-$ **0**.**00508**	0.00014	0.01501
HS	9.28	4	0.00431	0.00431	$-$0.00459	0.00014	0.01539
RM	9.28	14	0.01509	0.01509	$-$0.00362	**0**.**00013**	0.02354
GARCH	9.28	5	0.00539	0.00539	$-$0.00404	**0**.**00013**	**0**.**01494**
BiGAN	9.28	8	0.00862	0.00862	$-$0.00404	0.00014	0.01814
MC	9.28	7	0.00754	0.00754	$-$0.00416	0.00014	0.01738

Values in bold indicate the model(s) with the best performance per evaluation metric (column)

**Table 9:** Coverage and independence tests of VaR$^{99\%}$ models in EURUSD series

Model	$\text {LR}_{\text {uc}}$	$\text {LR}_{\text {ind}}$	$\text {LR}_{\text {cc}}$	DQ
DeepVaR	$$8.463^{**}$$ [0.004]	0.009 [0.926]	$$8.472^*$$ [0.014]	5.786 [0.448]
HS	3.846 [0.05]	0.035 [0.852]	3.881 [0.144]	$$29.349^{**}$$ [0.0]
RM	2.108 [0.147]	0.429 [0.512]	2.537 [0.281]	9.598 [0.143]
GARCH	2.386 [0.122]	0.054 [0.816]	2.441 [0.295]	$$22.326^{**}$$ [0.001]
BiGAN	0.184 [0.668]	0.139 [0.709]	0.324 [0.851]	$$26.285^{**}$$ [0.0]
MC	0.613 [0.434]	0.107 [0.744]	0.72 [0.698]	$$28.095^{**}$$ [0.0]

The *p* values are in brackets

All the aforementioned results, for each of the FX time-series, could be explained by the individual characteristics of the examined models, with the adaptation of each model to the returns’ volatility being the most influential. The VaR models that fit closer to the true returns would potentially suffer from VaR breaches, while the ones that generalize better would produce a greater firm loss (Sect. 3.2.3).

More specifically, as stated in Sect. 3.2.1, the HS model uses the last 1000 historical returns of the input time-series to estimate VaR. As a result, in order for the HS to alter to a stricter estimation, the most recent daily returns would have to be lower than the worst 10 (for 99% confidence interval) of the 1000-day historical window. Using such a large history, HS is able to capture most of the negative returns. However, in case of a sudden and permanent change in volatility, the adaptation of this model to the new inputs would be slow. This is also evident in Figs. 3, 4, 5 and 6 with HS being a straight line for long periods of time. Similar are the findings for the MC model as its input parameters (i.e., $\mu , \sigma ^2$) have been derived from the last 900 historical data.

As for the BiGAN model, Figs. 3, 4, 5 and 6 illustrate that the estimated VaR oscillates heavily between a short range of values as the generated distributions slightly variate between each day of the testing period. However, BiGAN fails to capture the sudden negative returns, indicating that this model could not efficiently predict VaR in the case of rare financial events.

On the other hand, the other two baseline models are both GARCH(1,1) type which are capable of capturing the time-varying volatility of returns. Although, their main difference is the historical data used to estimate their parameters. The effective historical data for the RM is set to 74 days, while for the GARCH model is 900 (Sect. 3.2.1) containing information from a quite larger information set. This explains the fact that the RM prediction is closer to the true PnL than the GARCH and HS models, producing the most VaR violations, for all the examined time-series, among the utilized models.

This trade-off between VaR model validity and adaptation to time-series variance is mitigated by DeepVaR. The latter, being trained for a period of 900 days, predicts the parameters of the returns distribution based on the last 15 values (context length) of the input series. In such way, DeepVaR is able to both “memorize” past information during training and optimally capture the time-series volatility using recent data for parameters’ estimation.

## Multivariate VaR performance

Apart from the univariate evaluation of each VaR estimation method, the performance of each model was evaluated in a realistic multivariate perspective, hence in the context of 1000 random portfolios. These portfolios were created randomly by producing both positive and negative positions on the aforementioned FX assets, with the absolute sum of the positions(weights) to be equal to one. It should be mentioned that these portfolios have not any “hold” position, which means that every time all available capital is allocated. This approach is not far from the real-life portfolio management as it is a common strategy towards minimizing commission fees. In addition, no commission fees or extra charges were taken into consideration to simplify the evaluation schema, given that they could be modeled as a constant common term in every VaR model without affecting the results drastically. On the contrary, to compute the VaR of a portfolio, the correlation $\rho$ among the FX instruments should be taken into account (Longerstaey and Spencer 1996). In this case, the VaR of a portfolio for a given day can be estimated by Eq. 15

15 $$\begin{aligned} \text {VaR}^{\alpha }_p = \sqrt{\text {VRV}^T}, \end{aligned}$$

where *V* is a vector of the weighted VaR estimates per instrument $V = [w_1\text {VaR}^{\alpha }_1, w_2\text {VaR}^{\alpha }_2, w_3\text {VaR}^{\alpha }_3, w_4\text {VaR}^{\alpha }_4]$ and *R* is the correlation matrix of FX assets’ daily returns, with the last 125 daily returns of the assets to be taken into account for the calculation of *R* matrix

$$\begin{aligned} R = \begin{pmatrix} 1 &{} \rho _{1,2} &{} \rho _{1,2} &{} \rho _{1,4} \\ \rho _{2,1} &{} 1 &{} \rho _{2,3} &{} \rho _{2,4}\\ \rho _{3,1} &{} \rho _{3,2} &{} 1 &{} \rho _{3,4}\\ \rho _{4,1} &{} \rho _{4,2} &{} \rho _{4,3} &{} 1 \end{pmatrix}. \end{aligned}$$

Furthermore, for long positions ($w_i>0$) $\text {VaR}^{\alpha }_i$ is used, while for short positions ($w_i<0$) $\text {VaR}^{1-\alpha }_i$, where $\alpha$ the confidence probability of VaR estimation and *i* is the corresponding FX asset. The procedure of portfolio VaR estimation is summarized in Algorithm 2.

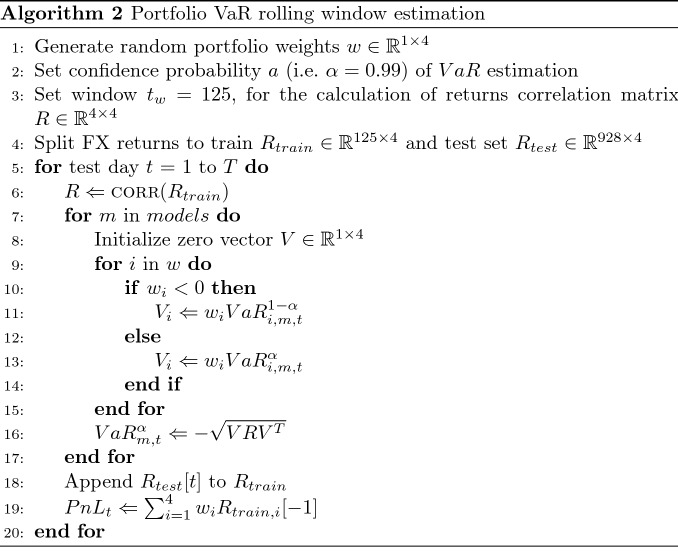



Finally, the average performance of each model over the random portfolios is summarized in Table 10. As presented in this table, DeepVaR achieved by far the lowest loss in all loss functions included, besides tick loss. Table 11 illustrates the percentage of the random portfolios per model passed the coverage and independence tests, depicting that DeepVaR is by far the most valid model.

**Table 10:** Average performance of VaR$^{99\%}$ models over the FX portfolios

Model	*E*[*v*]	*v*	$r_v$	$l_{\text {QL}}$	$l_Q$	$l_T$	$l_F$
DeepVaR	9.28	**2**.**90310**	**0**.**00319**	**0**.**00313**	$-$ **0**.**00427**	0.00011	**0**.**01351**
HS	9.28	7.29	0.00784	0.00784	$-$0.00361	0.00011	0.01618
RM	9.28	12.14	0.01308	0.01308	$-$0.00305	**0**.**00010**	0.02002
GARCH	9.28	8.61	0.00928	0.00928	$-$0.00321	**0**.**00010**	0.01660
BiGAN	9.28	11.51	0.01240	0.01240	$-$0.00320	0.00011	0.01966
MC	9.28	10.31	0.01111	0.01111	$-$0.00327	0.00011	0.01854

Values in bold indicate the model(s) with the best performance per evaluation metric (column)

**Table 11:** Percentage of portfolios passed the coverage and independence tests of VaR$^{99\%}$ per model in significant level 95%

Model	$\text {LR}_{\text {uc}}$	$\text {LR}_{\text {ind}}$	$\text {LR}_{\text {cc}}$	DQ
DeepVaR	72.8	**95**.**5**	**80**.**6**	**84**.**6**
HS	**76**.**9**	72.0	59.5	36.7
RM	65.2	95.3	68.3	55.9
GARCH	76.5	92.9	77.5	63.0
BiGAN	64.3	71.5	53.5	26.1
MC	70.4	68	54.8	26.7

Values in bold indicate the model(s) with the best performance per evaluation metric (column)

**Fig. 7:**
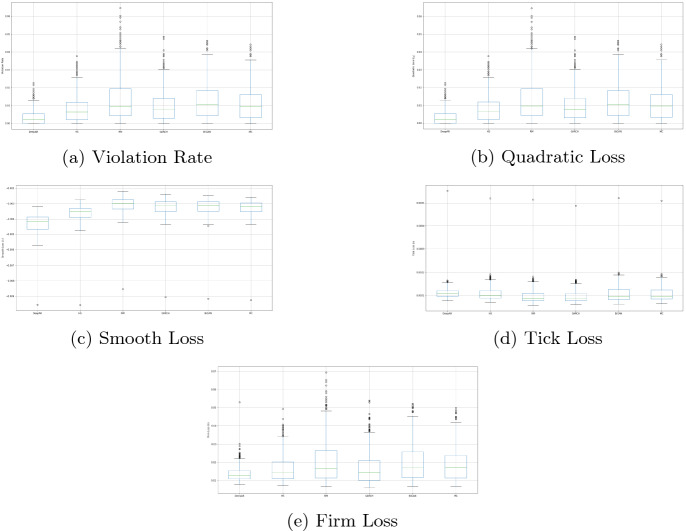
Box-plots of the $VaR^{99\%}$ performance per model over 1000 random portfolios. Each sub-figure refers to a different loss function. **a** and **b** Show that DeepVaR is the only model having violation rate and quadratic loss lower than 1-$\alpha$ (i.e., 0.01) confidence probability over most of the random portfolios. In terms of Smooth Loss (c), the superiority of DeepVaR over the rest of the models is evident. Tick loss (d) is the only metric where DeepVaR under-performs compared to the rest of the models. e Presents the results of VaR estimation firm loss, with DeepVaR to be the winning model

Moreover, the loss metrics are depicted in box-plots (Fig. 7) to provide a better overview of the VaR models’ performance over several random portfolios. Specifically, Fig. 7a shows that DeepVaR is the only model having a violation rate lower than $1-\alpha$ (i.e., 0.01) confidence probability over most of the portfolios. The violation rate of the other three models examined is highly dependent on the portfolio composition, while in some cases, the violation rate is significantly higher than the nominal threshold. Similar findings are derived from Quadratic Loss function (Fig. 7b).

In terms of Smooth Loss, which penalizes more heavily VaR violations, the superiority of DeepVaR over the rest of the models is evident (Fig. 7c). As far as the Tick Loss is concerned, this is the only metric where DeepVaR under-performs compared to the other models. However, DeepVaR has stable tick loss regardless of the portfolio, while in the other models, this metric has higher volatility among the different portfolios.

The last examined loss function, Firm Loss, takes into account the opportunity cost of capital, where firms would unnecessarily reserve capital according to the VaR estimates of their portfolios. Figure 7e presents the results of VaR estimation firm loss for all the portfolios, with DeepVaR to be again the winning model.

## Conclusions

This paper aims at addressing one of the main challenges in the financial sector, which is the constant search and development of more accurate risk estimation models. The utilization of Deep Neural Networks tools and techniques can introduce an innovative approach for VaR estimation, aiming towards a robust risk management framework.

To this direction, different parametric semi-parametric and non-parametric VaR estimation approaches were incorporated as baseline models, while an innovative framework based on a probabilistic Deep Neural Network was analyzed and compared against the baseline ones. The proposed framework yields better results in terms of the utilized evaluation metrics. More specifically, it is more effective than the others in terms of VaR violations and excess loss beyond the VaR threshold, while at the same time, it permits financial institutions to reserve less capital on liquid assets compared to the classical approaches. Additionally, the framework has the capability to incorporate other VaR models through the framework’s modularity.

Future work will focus on improving the efficiency of the proposed probabilistic approach in terms of high-frequency trading. To achieve the latter, intra-day data should be leveraged along with parallel and distributed computing techniques. It should also be highlighted that the hyper-parameter tuning of any deep neural network is a highly time-consuming task requiring extensive computational resources (Diaz et al. 2017). As a result, only a specific range of them, based on randomized grid search of the hyper-parameter space, was tested and evaluated in the context of our research, while further improvement of the model performance may be achieved through further hyper-parameters tuning. Finally, additional sources of complementary information could be integrated for improved results, such as sentiment analysis on texts (i.e., tweets and financial news) which may have an impact on the market movements.

## Supplementary Information

Below is the link to the electronic supplementary material.

Supplementary file1 (PDF 291 KB)

## Footnotes

1 https://github.com/hamaadshah/market_risk_gan_tensorflow.
