# Supplementary material for: DeepVaR: a framework for portfolio risk assessment leveraging probabilistic deep neural networks
Source: Digit Finance. 2022 Apr 13;5(1):29–56. doi: 10.1007/s42521-022-00050-0 (PMC9006212; doi:10.1007/s42521-022-00050-0)
Supplement: Supplementary file 1 — Supplementary file1 (PDF 291 KB) [file 42521_2022_50_MOESM1_ESM.pdf]

Appendix A Supplementary Information

Towards providing a clearer view of the added-value of the proposed framework, this section provides a comparison between DeepVaR and the best baseline model, GARCH, in terms of model's prediction accuracy and VaR performance in rare financial events.

As far as, the model's prediction accuracy is concerned, the deviation of the predicted asset returns $\hat{r}_{i,t}$ from true returns $r_{i,t}$ needs to be quantified. Thus, the estimated distributions of the parameterized DeepAR, leveraged by the DeepVaR framework, are evaluated in terms of continuous ranked probability score (CRPS) which can be interpreted as a generalized version of the mean absolute error (MAE) applicable in probabilistic forecasts Gneiting, Raftery, Balabdaoui, and Westveld (2004), and root mean square error (RMSE). The mathematical formulas to calculate the CRPS of DeepVaR and GARCH for a single prediction are provided by Eq. A1 and Eq. A2, respectively, while the RMSE is given by Eq. A3. As seen in Tables A1-A4, illustrating the statistics of CRPS and the RMSE of each time-series i in the testing period from 2018/01/01 to 2020/12/18, the DeepVAR achieved a better prediction accuracy than the GARCH model.

$$CRPS_D(F, r) = \int_z (F(z) - H(z - r))^2 dz \tag{A1}$$

where $F(x) = \int_{z \leq x} p(z)dz$ is the cumulative distribution function (CDF) of the DeepVaR forecast distribution F and $H(x)$ denotes the Heaviside step function, where r is the true observation Gneiting, Raftery, Westveld III, and Goldman (2005).

$$CRPS_G(\mathcal{N}(\mu, \sigma^2), r) = \sigma \left(\frac{r - \mu}{\sigma} \left(2\Phi \left(\frac{r - \mu}{\sigma} \right) - 1 \right) + 2\phi \left(\frac{r - \mu}{\sigma} \right) - \frac{1}{\sqrt{\pi}} \right) \tag{A2}$$

where μ and σ^2 are obtained from the GARCH model, r is the true observation, $\phi\left(\frac{r-\mu}{\sigma}\right), \Phi\left(\frac{r-\mu}{\sigma}\right)$ the PDF and the CDF, respectively, of the normal distribution with mean 0 and variance 1 evaluated at the normalized prediction error, $(r - \mu)/\sigma$ Gneiting et al. (2005).

$$RMSE(r - \hat{r}) = \frac{1}{N_{test}} \sum_{t=0}^{N_{test}-1} (r_t - \hat{r}_t)^2 \tag{A3}$$

Furthermore, the added-value of the DeepVaR approach in VaR estimation is evident during the period of increased volatility in financial markets during March 2020 due to the Covid pandemic. This fact is illustrated in Figure A1, comparing the daily VaR estimations of the two models among the four utilized time series (i.e., AUDUSD, GBPUSD, USDJPY, EURJPY). $VaR^{99\%}$ performance per model. In each sub-figure, the VaR estimation of each model is depicted against the true PnL (green and yellow dots, for positive and negative

Table A1: Prediction Accuracy on AUDUSD series. Mean, median and variance of the continuous ranked probability score (CRPS) and the root mean square error (RMSE) of each model in the testing period from 2018/01/01 to 2020/12/18. Lower values (in bold) are better.

Model	CRPS mean	CRPS median	CRPS variance	RMSE
DeepVaR	**0.002998**	**0.001855**	**0.000009**	**0.00564**
GARCH	0.004230	0.002139	0.000051	**0.00564**

Table A2: Prediction Accuracy on GBPUSD series. Mean, median and variance of the continuous ranked probability score (CRPS) and the root mean square error (RMSE) of each model in the testing period from 2018/01/01 to 2020/12/18. Lower values (in bold) are better.

Model	CRPS mean	CRPS median	CRPS variance	RMSE
DeepVaR	**0.002848**	0.001832	**0.000008**	**0.00537**
GARCH	0.002868	**0.001824**	0.000009	0.00538

Table A3: Prediction Accuracy on USDJPY series. Mean, median and variance of the continuous ranked probability score (CRPS) and the root mean square error (RMSE) of each model in the testing period from 2018/01/01 to 2020/12/18. Lower values (in bold) are better.

Model	CRPS mean	CRPS median	CRPS variance	RMSE
DeepVaR	**0.002098**	**0.001338**	**0.000005**	**0.00408**
GARCH	0.005165	0.001435	0.000175	0.00410

returns, respectively). The coral dots are the VaR violations for the cases where only one of the two models fails to capture the true PnL, while the red dots indicate that both DeepVaR and GARCH failed. As shown in Figures A1a-A1d the DeepVaR has fewer VaR violations than the GARCH for the period 2020/03/01-2020/3/31 over all the major forex pairs. As a result, the risk exposure could be monitored more accurately leveraging the proposed framework.

Additionally, DeepVaR showcases very promising results when comparing its performance among the (1000) random portfolios - as these are described in Section 5. Figure A2 shows the percentage and the number of VaR violations per model in March 2020 for the 1000 random portfolios. In the 86.1% of portfolios, DeepVaR had no violations, while for the GARCH, this is the case only for the 35.1%. The maximum number of VaR violations in a single portfolio for the DeepVaR was four (in the 1.7% of portfolios). On the other hand,

Table A4: Prediction Accuracy on EURUSD series. Mean, median and variance of the continuous ranked probability score (CRPS) and the root mean square error (RMSE) of each model in the testing period from 2018/01/01 to 2020/12/18. Lower values (in bold) are better.

	CRPS			RMSE
Model	mean	median	variance	
DeepVaR	**0.00204**	**0.001376**	**0.000003**	**0.00384**
GARCH	0.00300	0.001504	0.000036	**0.00384**

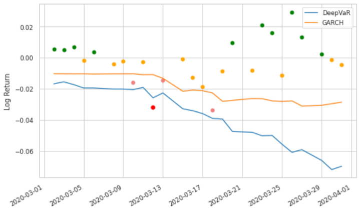

(a) AUDUSD

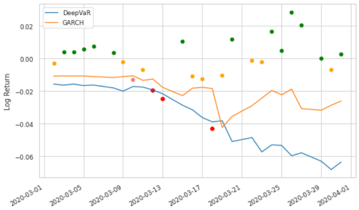

(b) GBPUSD

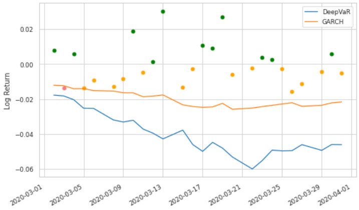

(c) USDJPY

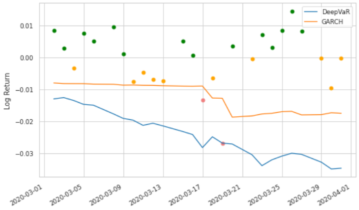

(d) EURUSD

Fig. A1: Daily $VaR^{99\%}$ estimations in March 2020 of DeepVaR and GARCH models among the four utilized time series (i.e., AUDUSD, GBPUSD, USDJPY, EURJPY). The VaR estimation of each model is depicted against the true PnL (green and yellow dots, for positive and negative returns, respectively). The coral dots are the VaR violations for the cases where only one of the two models fails to capture the true PnL, while the red dots indicate that both DeepVaR and GARCH failed. Sub-Figures A1a-A1d show that the GARCH model had from one to three more VaR violations than DeepVAR.

the 16.2% of portfolios had from four to seven violations when VaR estimation based on the GARCH model.

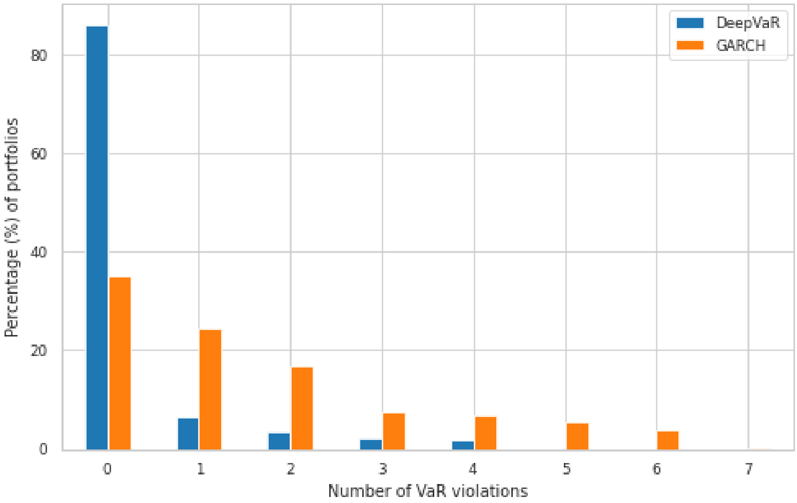

Fig. A2: Percentage and number VaR violations per model in March 2020 for 1000 random portfolios.
